# Reply to “Ensuring clinical translation of the Hernia ASCEND Hugo™ RAS training pathway: the need for outcome validation and anatomical accuracy”

**DOI:** 10.1007/s11701-025-02887-0

**Published:** 2025-10-16

**Authors:** Francesco Brucchi, Gianlorenzo Dionigi, Filip Muysoms

**Affiliations:** 1 University of Milan, Via Festa del Perdono 7, Milan, Italy; 2 Division of General and Endocrine Surgery, Istituto Auxologico Italiano, IRCCS (Istituto Di Ricovero E Cura a Carattere Scientifco), Milan, Italy; 3 Department of Pathophysiology and Transplantation, University of Milan, Milan, Italy; 4 Department of General Surgery, AZ Maria Middelares, Ghent, Belgium

Dear Editor,

We read with great interest the correspondence by Saleem et al. [1] regarding our article on the Hernia ASCEND Hugo™ RAS training pathway [2]. We are grateful for their insightful comments and for underscoring the key issues that are central to the future development of structured robotic hernia training.

## Clinical outcome validation

We fully concur that the ultimate validation of any training program must rest on patient outcomes. As highlighted both in our article and in our recent systematic review of robotic abdominal wall training models [3], the current evidence is fragmented and heterogeneous, with few studies linking curricula directly to operative outcomes. The ASCEND pathway, however, is built on the well-established principles of proficiency-based progression (PBP) training [4, 5]. In this model, all errors relevant to robotic hernia repair—such as mesh malposition, vascular injury, or nerve entrapment—are explicitly defined and recorded through validated scoring systems. A trainee can only progress once these errors are consistently avoided, and proficiency has been objectively demonstrated for the given procedure. This approach is not new: PBP has repeatedly been shown to reduce errors and improve technical performance across multiple domains of surgical education [6, 7]. For this reason, while the ASCEND paper did not (by design) present clinical outcome data, the effectiveness of the PBP principle itself does not need to be re-demonstrated. What remains essential is the multicenter correlation of Hernia ASCEND-specific milestones with clinical outcomes.

In this regard, early prospective data from our group on the transition from the da Vinci Xi to the Hugo™ RAS platform for r-TAPP showed no measurable learning curve in terms of operative time, with maintained safety and significant postoperative quality-of-life improvements [8]. These findings support the concept that structured, proficiency-based training enables efficient and safe adoption of new robotic platforms, reinforcing the translational potential of the ASCEND framework.

## Training models and anatomical fidelity

We acknowledge that porcine models, while providing unmatched live-tissue fidelity, cannot fully replicate human anatomical complexity, adhesions, or patient heterogeneity. This limitation is well recognized in the literature [3]. We acknowledge that porcine models, while providing unmatched live-tissue fidelity, cannot replicate the full complexity of human anatomy, adhesions, or patient heterogeneity. This limitation is well recognized in the literature. It is also true, however, that every training or simulation modality has intrinsic shortcomings when compared with real patients. The strength of the ASCEND pathway lies precisely in its *stepwise logic*: trainees must demonstrate proficiency at each level before progressing to the next, thereby reaching the patient only after validated competence in all preparatory stages. Moreover, as Saleem et al. note, real patients often present with obesity, adhesions, or other challenges; this is why, in our view, initial clinical cases must be deliberately simple, carefully selected, and always performed under the supervision of an experienced proctor. Complexity is then increased gradually, both in terms of patient characteristics and procedural difficulty, as outlined in the progressive adoption model described by Vierstraete et al. [9]. This approach balances the limitations of individual training models with the safety of a structured progression into clinical practice.

## Patient safety and non-technical skills

We also agree on the importance of embedding structured safety practices and non-technical skills. At present, however, the literature on non-technical skills specifically applied to robotic surgery remains scarce, and this represents a broader gap in surgical education [10].

The Hernia ASCEND pathway already incorporates emergency response, docking drills, and structured debriefing; nonetheless, we share the view that dedicated modules for decision-making, error prevention, communication, and crisis management are indispensable. These elements are aligned with recent advances in robotic surgical education and will be expanded in subsequent course versions.

In conclusion, we thank Saleem et al. for their constructive feedback. Their observations reinforce the rationale of ASCEND as a reproducible framework, while highlighting the priority areas for its validation and refinement. By integrating outcome-driven research, anatomically accurate training modalities, and enhanced safety curricula, the ASCEND pathway aims to contribute to the safe and standardized expansion of robotic hernia surgery worldwide.

## Data Availability

No datasets were generated or analyzed during the current study.
